# Uneven Expression of 20 Human Papillomavirus Genes Associated with Oropharyngeal Carcinoma

**DOI:** 10.5041/RMMJ.10508

**Published:** 2023-10-29

**Authors:** Ali Adel Dawood

**Affiliations:** Microbiology, Department of Anatomy, College of Medicine, University of Mosul, Mosul, Iraq

**Keywords:** Down-regulation, expression, gene, oropharyngeal carcinoma, up-regulation

## Abstract

**Background:**

Human papillomavirus (HPV) is considered to be responsible for 95% of virus-related cancers in many organs. Oropharyngeal carcinoma (OC) is distinguished by the transformation of the healthy epithelium into precancerous cells.

**Aim:**

The current study sought to examine the uneven gene expression of 20 genes among those scanned by microarray for oropharyngeal cancer patients.

**Materials and Methods:**

The GSE56142 dataset was extracted from the Gene Expression Omnibus of the National Center for Biotechnology Information; 24 specimens were evaluated. Gene ontology (GO), the Kyoto Encyclopedia of Genes and Genomes, and the protein–protein interaction (PPI) were used to depict the biological roles of the genes under investigation using types of software.

**Results:**

Six genes out of 20 in patients with invasive OC had a binding correlation with high expression (*PDGFRS*, *COL6A3*, *COL1A1*, *COL3A1*, *COL2A1*, and *COL4A1*), and only two genes with low expression (*CRCT1* and *KRT78*). The expression levels of 20 genes were examined for patients with OC versus head and neck squamous cell carcinoma (HNSCC). The correlation coefficient between highly expressed genes of the OC group was statistically significant at the *P*<0.05 level.

**Conclusions:**

High expression levels of specific genes may serve as diagnostic tumor markers, particularly in the early stages of cancer, and testing should be performed in OC and HNSCC patients.

## INTRODUCTION

Viruses are responsible for 15%–20% of all human cancers. The investigation of oncogenic viruses and how they target regulatory nodes has been essential for understanding the etiology of many human cancers.^1^ Human papillomavirus (HPV) has been linked to a dramatic increase in the incidence of mouth and throat cancers over the past 30 years. There are numerous forms of oropharyngeal carcinomas (OCs).^2^ The great majority are oropharyngeal squamous cell carcinomas (OSCC), which have two categories based on HPV testing. Tobacco and alcohol are common causes of HPV-unrelated cancers. Around 15,000 new cases of oropharyngeal cancer are diagnosed in the US annually, with the vast majority being HPV-positive.^3^

A significant number of studies have been conducted worldwide on HPV infection epidemiology as well as the carcinogenic qualities caused by various HPV genotypes. The HPV genome encodes both early (E1 to E8) and late (E9 to L) structural genes (L1 and L2). The structural proteins are produced in the late-coding regions, while the oncogenic E6 and E7 are primarily located in the early-coding regions.^4^,^5^

These tumors are autonomous biological structures, and HPV16 is believed to be responsible for 95% of malignancies related to viruses in several organs. Oropharyngeal carcinoma is characterized by the change of normal epithelium into precancerous tissue.^6^ Although the existence of HPV subtypes in invasive OC has been investigated in major epidemiological research, the prevalence of HPV subtypes remains unknown. However, it is known that OC rates have increased significantly among the male sex of white, Hispanic, and other races, whereas the incidence has decreased among black men.^7^,^8^

In light of the costliness of HPV vaccination, it has been argued that widespread vaccination of girls would lead to herd immunity and reduce the need to vaccinate boys.^9^ However, variations in vaccination uptake due to ongoing logistical, sociological, and cultural hurdles are likely to impede the ability of communities to achieve the required levels to prevent future HPV-related cancers.^10^,^11^

Several factors influence OC treatment, including the patient’s overall health, disease stage, tumor size and location, lymph node status, the patient’s ability to speak and swallow, and the extent of metastasis.^12^ Radiation therapy, chemotherapy, and surgery (including laparoscopic robotic surgery and neck dissection) are all viable options. Surgery is occasionally followed by radiation treatment. When surgery is not an option, combination therapy (radiation and chemotherapy) is most commonly used.^13^,^14^ Each patient deserves individualized care, and a multidisciplinary team can offer that. Researchers are exploring ways to lessen treatment intensity while maintaining the patient’s quality of life, for example by administering less intense courses of radiation, chemotherapy, or immunotherapy.^15^

The current study sought to examine the uneven gene expression of 20 genes among those scanned by microarray for oropharyngeal cancer patients and compared their expression in patients with head and neck squamous cell carcinoma (HNSCC).

## MATERIALS AND METHODS

Following the microarray experiment applied to oropharyngeal patients by Masterson et al.,^16^ the GSE56142 dataset was extracted from the Gene Expression Omnibus (GEO) of the National Center for Biotechnology Information.^16^ Individuals with primary OSCC were enrolled in the study. A total of 24 specimens were evaluated: 12 with normal epithelium (normal) and 12 with invasive OSCC. Table 1 depicts the distribution of patient profile information. The cancer grade of patients included in the study ranged from grade 3 to grade 4.

**Table 1:** Profile of All Participants in the Current Study.

Sample Accession Number	Age	Sex	State	Stage	HPV Status	Subsite
GSM1355616	48	Male	Normal	IV	+	Tongue base
GSM1356617	48	Male	Invasive	IV	+	Tongue base
GSM1356618	65	Male	Normal	IV	+	Tongue base
GSM1356619	65	Male	Invasive	IV	+	Tongue base
GSM1356620	52	Male	Normal	IV	+	Tonsil
GSM1356621	52	Male	Invasive	IV	+	Tonsil
GSM1356622	58	Male	Normal	IV	+	Tongue base
GSM1356623	58	Male	Invasive	IV	+	Tongue base
GSM1356624	75	Male	Normal	IV	+	Tonsil
GSM1356625	75	Male	Invasive	IV	+	Tonsil
GSM1356626	60	Female	Normal	IV	+	Tongue base
GSM1356627	60	Female	Invasive	IV	+	Tongue base
GSM1356628	43	Male	Normal	IV	−	Tongue base
GSM1356629	43	Male	Invasive	IV	−	Tongue base
GSM1356630	56	Male	Normal	IV	+	Tonsil
GSM1356631	56	Male	Invasive	IV	+	Tonsil
GSM1356632	59	Male	Normal	IV	+	Tongue base
GSM1356633	59	Male	Invasive	IV	+	Tongue base
GSM1356634	77	Male	Normal	III	+	Tongue base
GSM1356635	77	Male	Invasive	III	+	Tongue base
GSM1356636	55	Male	Normal	III	+	Tonsil
GSM1356637	55	Male	Invasive	III	+	Tonsil
GSM1356638	81	Male	Normal	III	−	Tonsil
GSM1356639	81	Male	Invasive	III	−	Tonsil

GSM, gene sample platform ID.

Tumor samples were evaluated for HPV status with p16INK4A expression by a histopathologist. The PGMY PCR technique was used for HPV16 DNA amplification of the samples. Whole transcriptome analysis of fresh tissue samples was performed utilizing the Illumina BeadArray (Illumina, San Diego, CA, USA), capable of assessing approximately 47,000 transcripts. Quantitative real-time PCR (qRT-PCR) confirmed the findings. To gain a deeper understanding of the transition from benign to malignant development in OC, the gene expression profiles of tumor samples were compared to site-matched normal epithelium controls.

The Gene Ontology (GO) and the Kyoto Encyclopedia of Genes and Genomes were used to depict the biological roles of the genes under investigation. Cytoscape software and a set of putative hub genes were used to build the protein–protein interaction (PPI). The GraphPad Prism 8.0 software confirmed five candidate hub genes in the dataset GSE56142. Analysis of the GPL10558 dataset enabled determination of the diagnostic value of each gene and display in a ROC curve. Gene Set Enrichment Analysis was used to investigate the roles of the genes.

Gene symbols were assigned to gene probes on the GPL10558 Illumina Human HT-12 V4.0 (Illumina, San Diego, CA, USA) expression bead-chip using data from microarray annotations. When a probe matched more than one gene symbol, a gene symbol was picked at random. Over 31,000 annotated genes were targeted using more than 47,323 probes derived from the National Center for Biotechnology Information (NCBI). Expression levels for identified genes were determined based on the aligned data by totaling the number of reads associated with all exons and splicing events for a given gene and then dividing that parameter value by the normalized number of mapped reads for that sample.

Patients were put into two groups: those with normal epithelium (Normal Group) and those with invasive OSCC (Invasive OSCC Group). Table 1 depicts the distribution of patient profile information. Analysis and estimation of group differences were performed using GEO2R. The GEO was used to help researchers find genes with varied expression depending on the type of experiment being conducted. The top 250 genes were identified, and their *P*-values used to rank them in Table 2, which summarizes the study findings. The *P*-value for these genes was much lower than that of the others.

**Table 2:** Description of 20 Selected Genes and Their IDs.

Gene Symbol	Gene Title	ID	Adj. *P* Value	*P* Value	*t*	B	logFC
***PDGFRB***	Platelet-derived growth factor receptor beta	ILMN_1815057	0.017	<0.001	−5.22	2.675	−1.015
***TEAD2***	TEA domain transcription factor 2	ILMN_1682781	0.005	<0.001	−6.67	5.868	−1.173
***COL1A2***	Collagen type I alpha 2 chain	ILMN_2104356	0.005	<0.001	−6.56	5.625	−2.238
***COL1A1***	Collagen type I alpha 1 chain	ILMN_1701308	0.005	<0.001	−6.51	5.526	−2.419
***RCN1***	Reticulocalbin 1	ILMN_1800276	0.006	<0.001	−6.21	4.882	−0.588
***COL3A1***	Collagen type III alpha 1 chain	ILMN_1773079	0.007	<0.001	−6.14	4.718	−1.679
***COL6A3***	Collagen type VI alpha 3 chain	ILMN_2307861	0.007	<0.001	−6.11	4.672	−1.524
***IFI6***	Interferon alpha inducible protein 6	ILMN_1687384	0.024	<0.001	−4.58	1.213	−1.75
***COL4A1***	Collagen type IV alpha 1 chain	ILMN_1653028	0.016	<0.001	−5.4	3.085	−1.978
***CERCAM***	Cerebral endothelial cell adhesion molecule	ILMN_1750563	0.016	<0.001	−5.39	3.053	−0.255
***NDRG2***	NDRG family member 2	ILMN_2361603	0.017	<0.001	5.26	2.779	1.632
***FAM3D***	Family with sequence similarity 3 member D	ILMN_1720433	0.022	<0.001	4.85	1.836	2.17
***KRT78***	Keratin 78	ILMN_1737653	0.023	<0.001	4.72	1.525	2.901
***SLURP1***	Secreted LY6/PLAUR domain containing 1	ILMN_1705080	0.025	<0.001	4.43	0.863	3.028
***MUC21***	Mucin 21, cell surface associated	ILMN_1700978	0.026	<0.001	4.34	0.661	2.56
***OSBPL10***	Oxysterol-binding protein-like 10	ILMN_1669497	0.006	<0.001	6.26	4.992	0.758
***TYRO3***	TYRO3 protein tyrosine kinase	ILMN_1740169	0.012	<0.001	5.72	3.799	0.967
***CHCHD10***	Coiled-coil-helix-coiled-coil-helix domain containing 10	ILMN_1740170	0.0141	<0.001	5.54	3.41	0.619
***RNASE7***	Ribonuclease A family member 7	ILMN_1712849	0.005	<0.001	6.77	6.06	2.261
***CRCT1***	Cysteine-rich C-terminal 1	ILMN_1803452	0.022	<0.001	4.9	1.951	2.738

Adj., adjusted; B, bucket testing; logFC, log-fold change; ILMN, luminal A and Her2-enriched tumors against controls; *t*, statistical *t*-test.

Blue genes: down-regulation expression; Red genes: up-regulation expression.

To address multiple testing issues and account for the possibility of false positives, adjusted *P-*values were obtained using the conservative Bonferroni correction method. This methodology involved multiplying the raw *P*-values by the total number of gene tests conducted. By adjusting the *P*-values in this manner, the significance threshold for each individual test is more stringent, helping to control the overall type 1 error rate across multiple comparisons.

A total of 250 genes were filtered down to 20, with the expression levels of all samples being analyzed. A free internet-based STRING program (STRING CONSORTIUM 2023: https://string-db.org) was used to determine how closely related the genes were. The iTOL and STRING servers were employed to obtain the genealogical tree. The iDEP.96 software (http://bioinformatics.sdstate.edu/idep96/) was used to get the heat map of the expressed genes. The heat map of the selected gene was extracted using UALCAN software (https://ualcan.path.uab.edu) according to the HNSCC tissue pattern. The expression of chosen genes in OC patients was compared to that of patients with HNSCC, utilizing oncology data subset sessions of the UALCAN software.

## RESULTS

**Figure 1:**
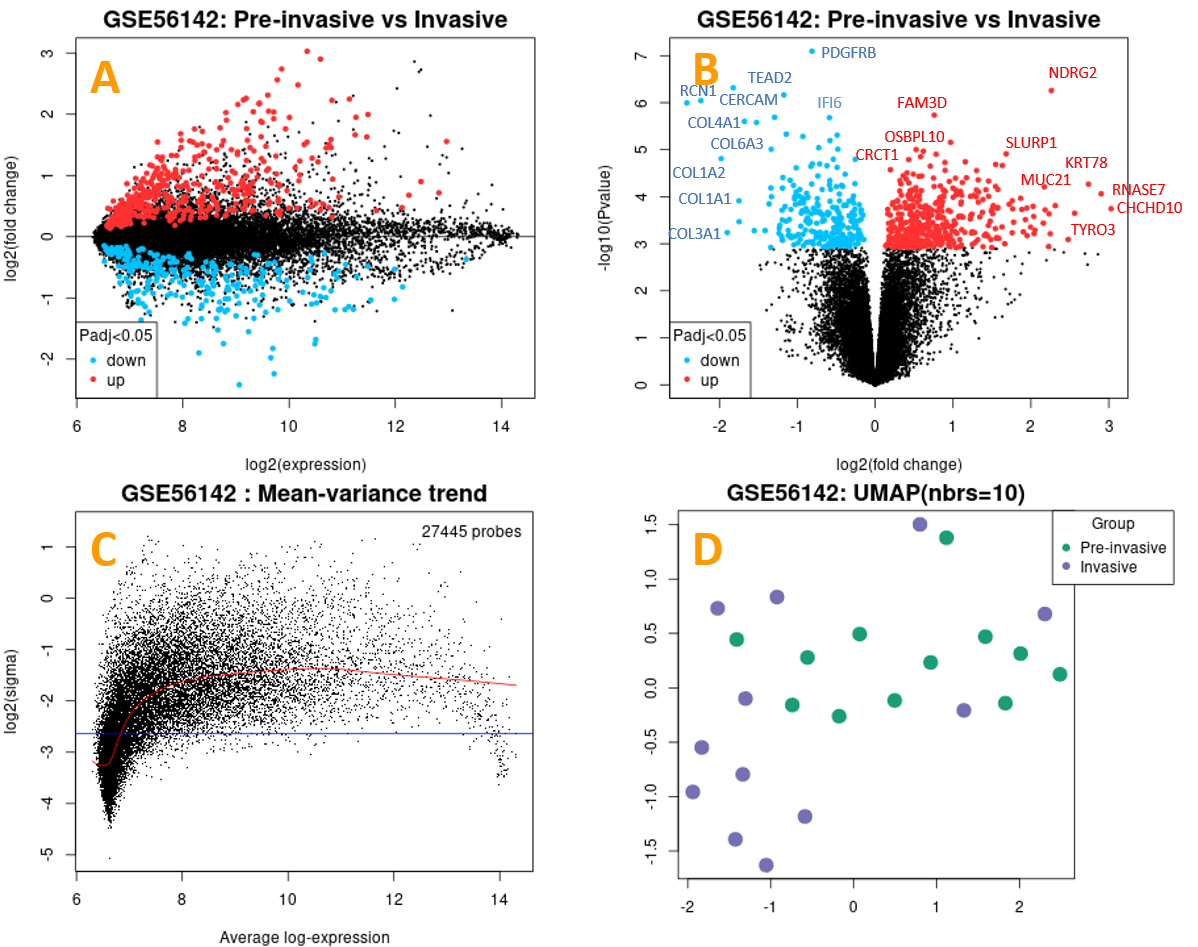
Depictions of Genes with Altered Expression. **A:** Volcano plot showing the prevalence of genes between the normal and invasive OSCC groups. Black dots, normal gene expression; blue dots, down-regulation; red dots, up-regulation at log_2_. **B:** Volcano plot showing high variance distribution between expression genes at log_10_. (Colors same as in panel A.) **C:** Average log expression of 27,445 probes: above red line are up-regulation genes; below the blue line are down-regulation genes. Black dots mark distribution of genes. **D:** A UMAP plot presenting sample distribution by color. Green dots, normal group; purple dots, invasive OSCC group.

The Supplement provides data regarding the expression of 250 genes in 24 samples. Table 2 displays the adjusted *P*-value, *P*-value, logFC (log-fold change), *t*-test, and B-test (bucket testing) results for the 20 genes with the greatest expression variation. A volcano plot (Figure 1A, B) illustrates differentially expressed genes by plotting statistical (−log_10_ *P-*value) versus magnitude of change (log_2_ fold change).

Normal gene expression for the GSE56142 cohort is shown in Figure 1A. At log_2_ with adjusted *P*<0.05, the black dots reflect steady expression near zero, the blue dots denote down-regulation, and the red dots denote up-regulation. The number of neighbors used in the analysis is displayed within the graph. Figure 1B at log_10_ with adjusted *P*<0.05 demonstrates no statistically significant overlap between the patient groups concerning the variation in gene expression.

Figure 1C illustrates the average log expression and mean-variance pattern across 27,445 probes. The distribution width can serve as a measure to determine whether the selected samples are suitable for expression analysis. The scattered nature of the patient sample values indicates that the data have been standardized and are mutually comparable. The implication from this figure is that value distribution of all selected samples was equal, enabling meaningful comparisons and analyses. To better see the connections between various samples, the uniform manifold approximation and projection (UMAP) method was used to reduce the number of dimensions. Figure 1D shows the data after log transformation and normalization.

**Figure 2:**
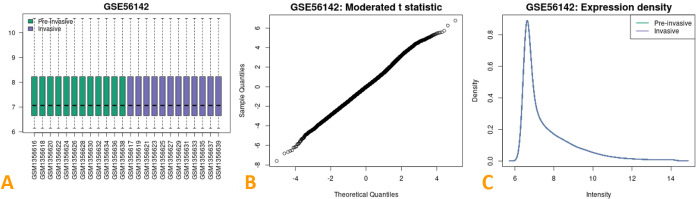
Expression of Microarray Scanned Genes. **A:** Box plot of patient profiles reflects the standardization and comparability of the data. **B:** Limma plot: Dataset arrangement according to the theoretical quantiles of the *t*-test distribution. **C:** Curve of the gene expression density.

A box plot was used to differentiate between selected sample values (Figure 2A). The concentration of patient sample values around the median suggests that the data were standardized and mutually comparable, indicating that all selected samples exhibited the same distribution of values, making them equivalent for analysis purposes. Figure 2A illustrates log transformation and normalization of the data. Figure 2B demonstrates the dots perfectly aligned in a straight line, indicating that the observed values aligned with the theoretical predictions, providing evidence of the validity and reliability of the test results. Note that gene expression intensity increased at the value of 7, at which point the curve steepened (Figure 2C).

A subgroup of 20 genes was chosen based on their varying levels of gene expression, specifically focusing on the presence of high and low gene expression. These genes were selected in order to investigate their potential involvement in the context of HPV infection. However, it is also possible to include additional genes for further analysis to explore the impact of HPV on a broader range of genes (Supplement). Table 2 displays the statistical variations in gene expression based on LogFC, adjusted p-values, and p-values. The genes were most abundant and variant in terms of logFC, B, *t*, *P*, and adjusted *P-*values.

**Figure 3:**
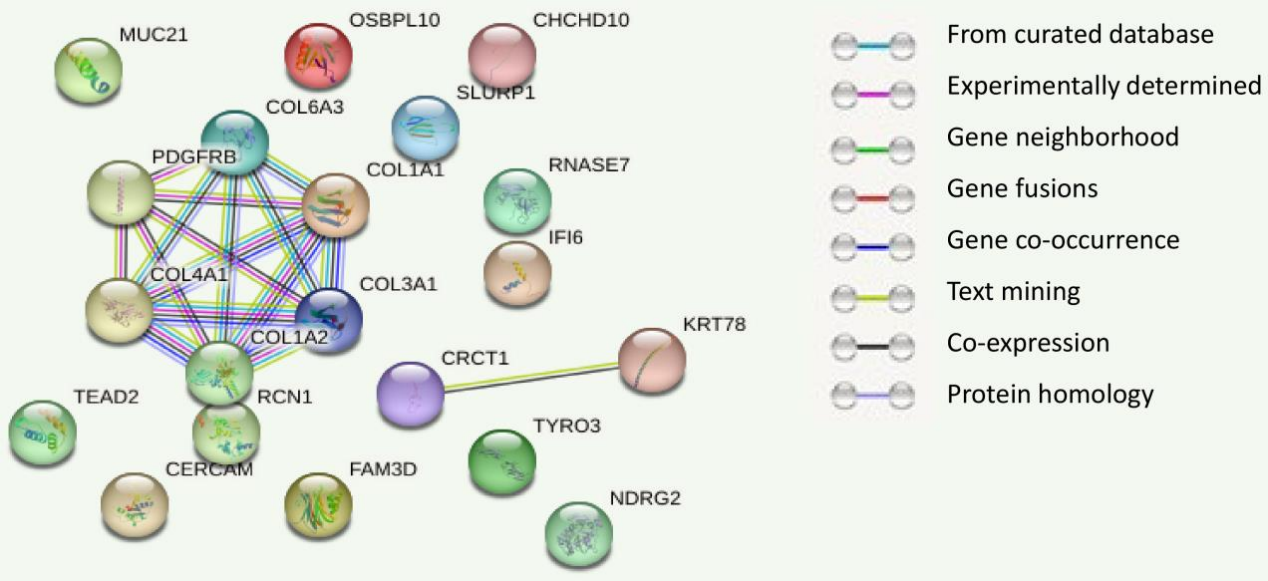
Two Sets of Genes Showing Multi-interactions. **First group:** *PDGFRB*, *COL6A3*, *COL1A1*, *COL3A1*, *COL1A2*, and *COL4A1*. **Second group:** *CRCT1* and *KRT78*.

Analyzing the protein–protein interaction of the 20 genes revealed two groups with binding associations. The first group had six genes (*PDGFRS*, *COL6A3*, *COL1A1*, *COL3A1*, *COL2A1*, and *COL4A1*) with down-regulated gene expression. The second group (*CRCT1* and *KRT78*) had up-regulated gene expression (Figure 3). Furthermore, the first group of genes were interconnected through various mechanisms such as gene fusion, text-mining analysis, co-expression, and translation into homology proteins. On the other hand, the second group of genes were connected in only two ways: co-expression and text-mining (text analytics between 2 proteins). These findings suggest that the genes linked to OCs are the outcome of gene expression.

**Figure 4:**
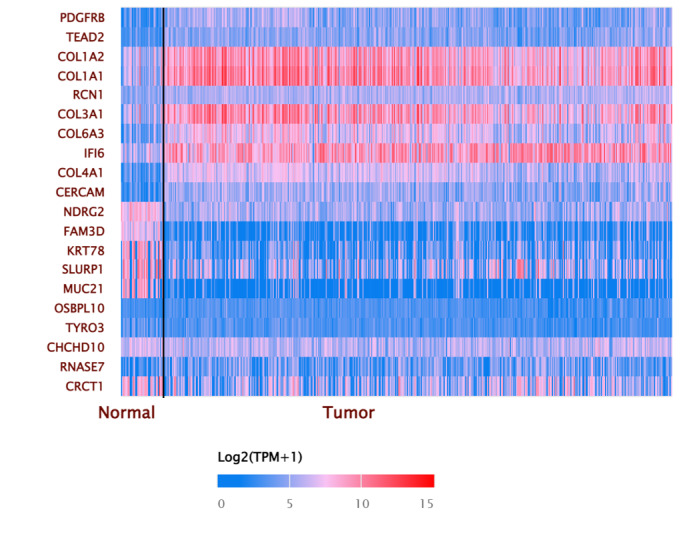
Heat Map of the Expression Pattern of Input Selected Genes in the Head and Neck Squamous Cell Carcinoma.

Figure 4 presents a heat map for the 20 selected HNSCC genes. Note that certain genes were rendered with a prominent pink color, specifically *COL1A2*, *COL1A1*, *COL3A1*, *COL6A3*, and *IF16*, indicating a high level of expression, ranging from 10 to 15 at log_2_ scale. It is important to note that the intensity of the pink color corresponds to the magnitude of expression, with stronger shades representing higher levels of gene expression.

**Figure 5:**
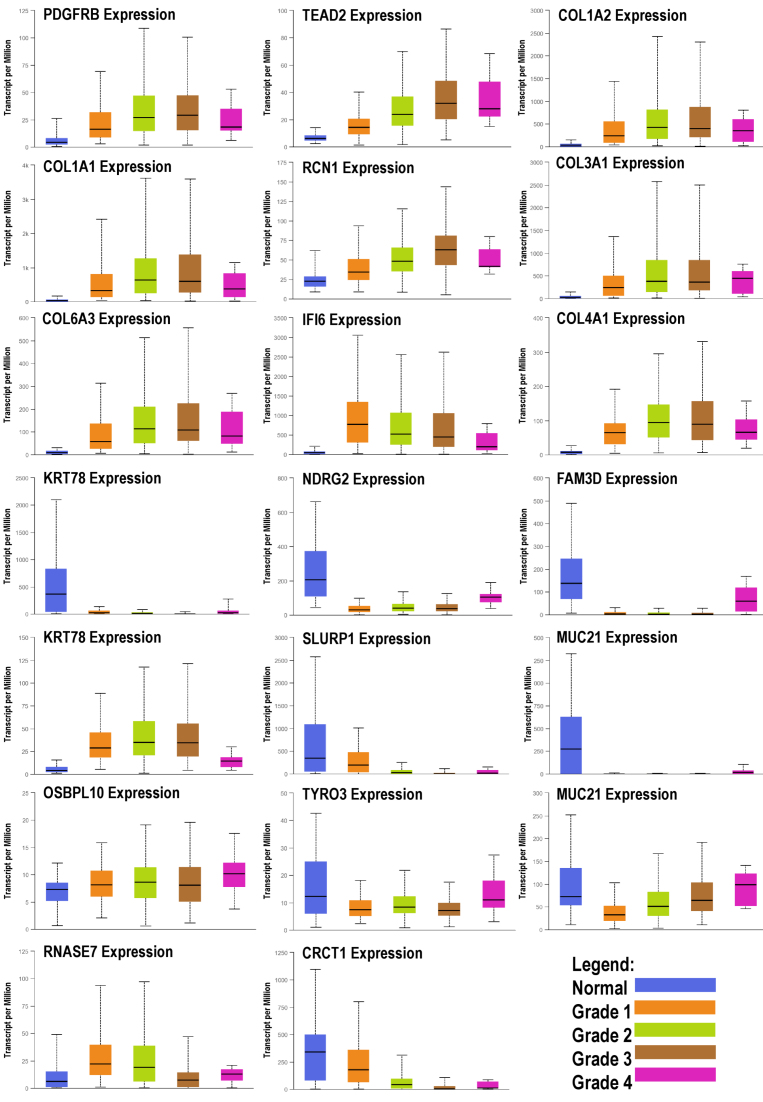
Box Plots of Selected Expression Genes of Head and Neck Squamous Cell Carcinoma (HNSCC) in the Four Grade Stages Compared to Normal.

Patients with HNSCC were exposed to the designated genes. The expression of these genes was detected, and it was determined that their expression differed from that of OC patients. Gene expression levels for all four tumor stages are depicted in Figure 5. According to our study findings, at the same level of gene expression analysis, patients diagnosed with HNSCC showed no significant differences in cancer grade for the genes *PDGFRB*, *TEAD2*, *COL1A2*, *RCN1*, *OSBPL10*, and *CHCHD10*. The *P*-value analysis, set at a significance level of *P*<0.05, indicated that these genes had comparable cancer grades among the HNSCC patients.

**Figure 6:**
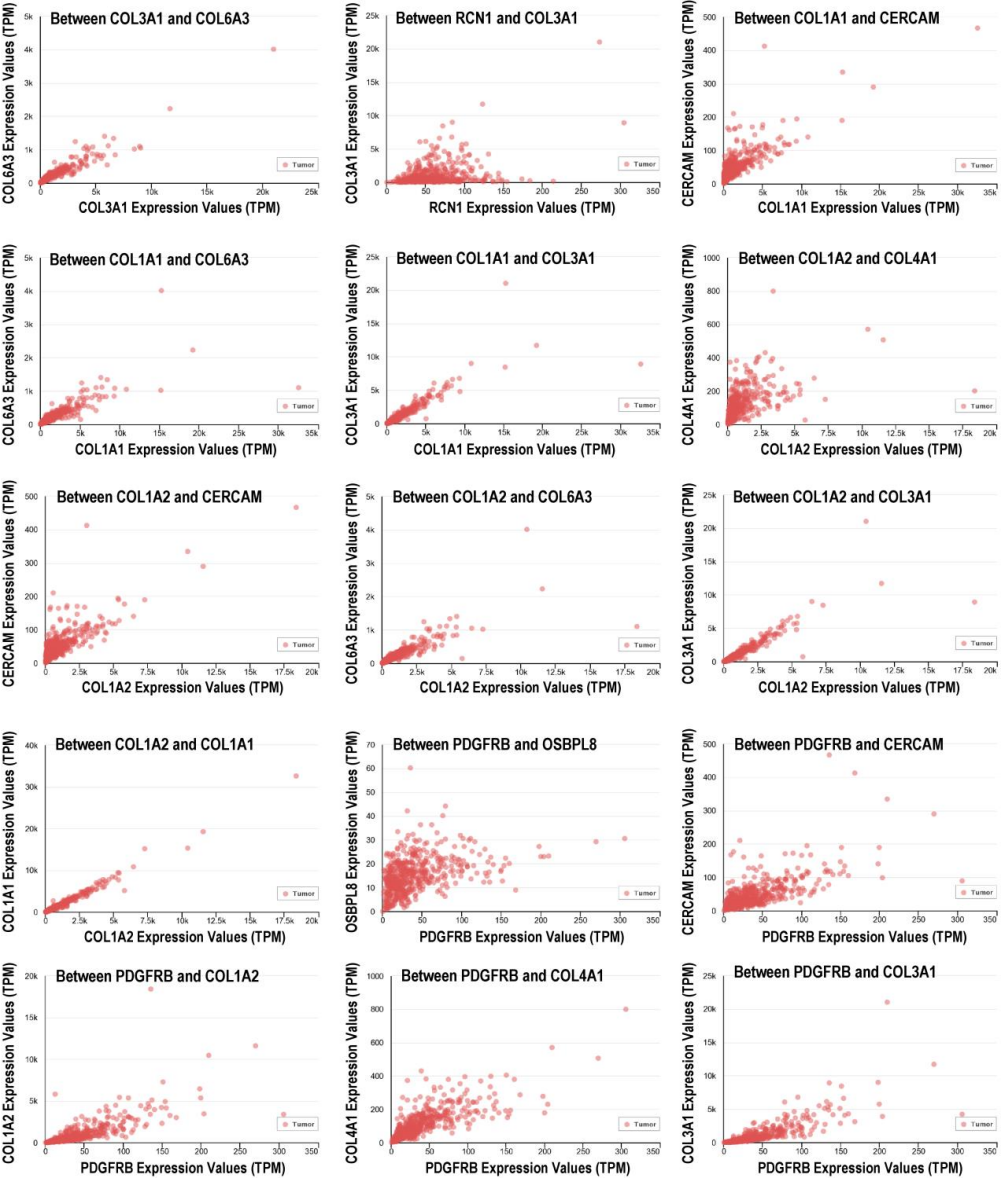
Gene Expression Correlation between Four Types of Selected Genes in Head and Neck Squamous Cell Carcinoma (HNSCC) and a Value of Tumor Progression Metastasis (TPM).

When analyzing the expression of the chosen genes in HNSCC patients using Pearson’s statistical software, strong differential correlations (in the range 78%–96%) and the value of tumor progression and metastasis were observed at *P*<0.05 (Figure 6). The correlation coefficient is in the range 0.68–0.89.

## DISCUSSION

Between 15% and 20% of all human malignancies are caused by viruses. Several viruses play significant roles in the multi-stage development of malignant tumors.^17^ The prevalence of human papillomavirus infection and the carcinogenic features of various HPV genotypes have been the subject of several studies around the world. Previous research had shown that HPV16 was present in around 25% of oropharyngeal dysplastic lesions.^18^ Because some studies included OC lesions, the same study concluded that this was an underestimation (a subsite that generally has a low frequency of HPV16). Most patients with HPV+OSCC (about 75%) present at a late stage (stage III or IV) because of cystic nodal disease.^19^

The Oropharyngeal Cohort Study differentiated OC based on the presence or absence of HPV (as indicated by p16 overexpression). Due to the revisions made in the approach to N staging, specifically in the context of HPV-positive disease, a significant number of individuals were reclassified to a lower stage. These revisions likely resulted in the downgrading of the disease stage for many individuals with HPV-positive disease. Furthermore, unlike traditional staging systems, which classed locally progressed cancer as stage IVa, this update solely uses the term “stage IV” for metastatic disease. Among other things, these advancements have enabled better OC discrimination, which is especially important during the de-intensification phase of therapy.^20^,^21^ The ability to accurately distinguish between different stage groups, such as stages II, III, III, and IV, may be uncertain due to overlapping results observed in specific individuals. This means that patients at a certain stage may exhibit similar characteristics as those at a later stage. Consequently, it becomes necessary to adapt the staging approach in clinical practice by incorporating other relevant prognostic markers. These additional markers can provide valuable insights and assist in refining the staging system to improve the accuracy of prognosis and treatment planning for patients.^22^

Out of more than 47,000 genes, 250 were chosen for this investigation based on their highest estimated expression in patient samples. A subset of these genes (20) was chosen because of their abnormally excessive expression levels compared to normal genes. Invasive tissue samples showed high levels of many genes (*CERCAM*, *COL1A1*, *COL1A2*, *COL6A3*, *COL4A1*, *COL3A1*, *IF16*, *PDGFRB*, *RCN1*, and *TEAD2*), whereas low levels were seen for other genes (*CHCHD10*, *CRCT1*, *KRT78*, *TYRC3*, *NDRG2*, *MUC21*, *SLURP1*, and *RNASE*7). There was no difference in the *FAM3D* and *OSBPL10* gene expression between the two groups (OC and HNSCC).

By studying the protein interaction, our results verified that six genes with high gene expression were closely associated as a set of unified genes with systemic expression, specifically *COL1A1*, *COL1A2*, *COL6A3*, *COL4A1*, *COL3A1*, and *PDGFRB*. The high expression of these genes presented and contrasted clearly in the heat map analysis of tumor tissue in Figure 4.

Since our study comprised individuals in stages III and IV of OC cancer, genes from the same stages were compared to HNSCC patients. Most of the genes showed an increase in gene expression during the stages of cancer, particularly in the last two stages, except for *NDRG2*, *FAM3D*, *KRT78*, *SLURP1*, *MUC21*, and *CRCT1*, which showed a significant drop in gene expression compared to normal tissue. These findings are consistent with the expression data of the chosen genes in OC patients.

Our findings indicate that the related genes with higher expression in invasive tissues have the greatest influence and can be employed as markers for OC in the field of diagnostics and tumor-grade classification. The expression levels of these genes were examined between patients with OC and HNSCC (Figure 6). The correlation coefficient between highly expressed genes was statistically significant (*P*<0.05).

Our study identified certain genes that play a crucial role in the development of specific types of cancer. The dysregulation of these genes serves as an indication or marker for the onset of various cancers. For instance, previous studies have shown a correlation between the expression of collagen type 1 genes and the occurrence of oral, gastric, and bone metastatic cancers.^23^–^26^ Another gene, *KRT78*, has been identified as a biomarker and is associated with oral pre-cancer.^27^

The recent identification of a gene expression profile associated with a poor prognosis in patients with HPV-OC and its similarities with HPV-HNSCC supports the observations made in the current study. This finding suggests that there may be common molecular features and underlying mechanisms between these two types of cancers, contributing to a worse prognosis in affected individuals. The discovery of this gene expression profile provides valuable insights into potential molecular drivers of aggressive disease and can have implications for personalized treatment strategies and prognostic evaluations in HPV-associated cancers.^23^ In light of these findings, it will be essential to identify whether or not cells produced from recurrent HPV-OCs are as dependent on continuous viral oncogene expression as those derived from initial tumors. If not, this could have consequences for the efficacy of HPV-targeted therapy, such as therapeutic vaccinations, in individuals with advanced illness.

## CONCLUSIONS

Papillomavirus is one of the most lethal cancer-causing viruses. Due to the lack of viable cancer treatment, monitoring changes in the expression of tumor-affected genes is crucial. Our study revealed that six highly expressed genes are closely associated and have a discernible effect on invasive tumor patients. It is essential to consider the high expression of certain genes as diagnostic tumor indicators, especially in the early-stage cancers.

## Supplementary Information



## Abbreviations

GEO: Gene Expression Omnibus
GO: gene ontology
HNSCC: head and neck squamous cell carcinoma
HPV: human papillomavirus
NCBI: National Center for Biotechnology Information
OC(s): oropharyngeal carcinoma(s)
OSCC: oropharyngeal squamous cell carcinoma(s)
PPI: protein–protein interaction
qRT-PCR: quantitative real-time PCR
