# Supplementary material for: Uneven Expression of 20 Human Papillomavirus Genes Associated with Oropharyngeal Carcinoma
Source: Rambam Maimonides Med J. 2023 Oct 29;14(4):e0020. doi: 10.5041/RMMJ.10508 (PMC10619986; doi:10.5041/RMMJ.10508)
Supplement: Supplementary file 1 [file rmmj-14-4-e0020-Supplement.pdf]

SUPPLEMENTARY MATERIAL

*This appendix has been provided by the authors for the benefit of readers*

# Supplement to Uneven Expression of 20 Human Papillomavirus Genes Associated with Oropharyngeal Carcinoma

**Dawood AA. Uneven Expression of 20 Human Papillomavirus Genes Associated with Oropharyngeal Carcinoma. Rambam Maimonides Med J 2023;14 (4):e0020. doi:10.5041/RMMJ.10508**

## EXPRESSION LEVELS OF THE 20 INVESTIGATED PROFILE GENES VARY CONSIDERABLY BETWEEN THE 2 PATIENT GROUPS

### *CERCAM*

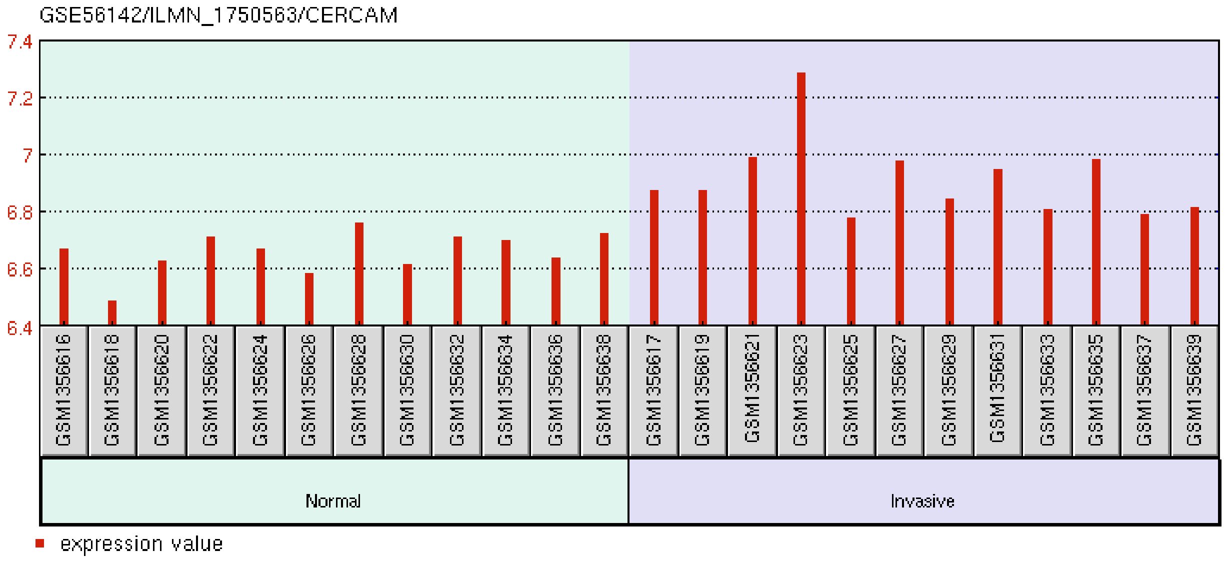

## *CHCHD10*

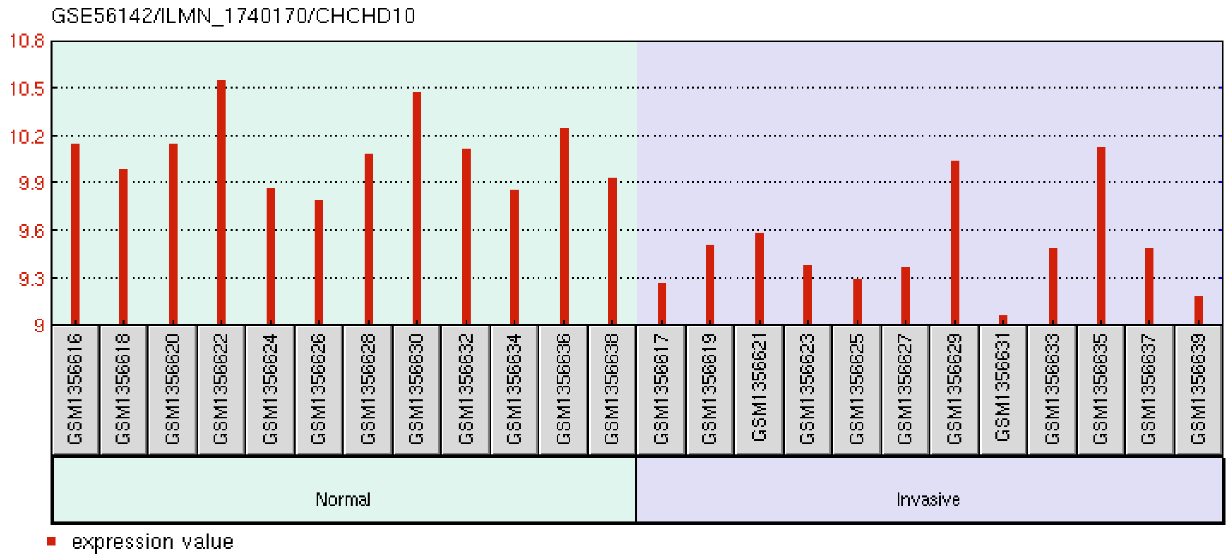


## *COL1A1*

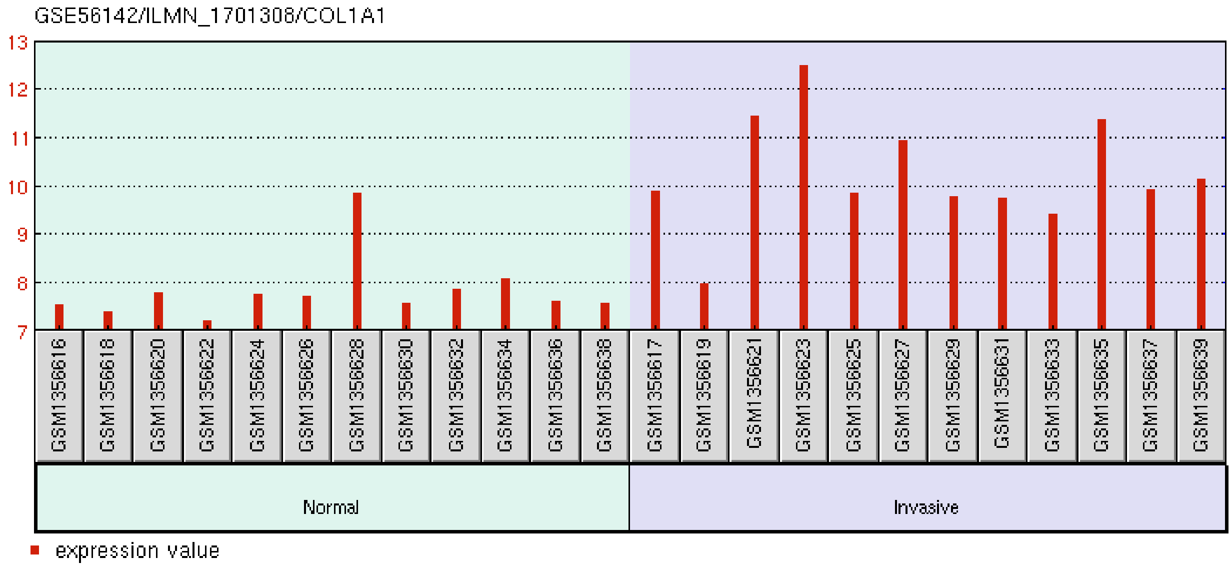

## *COL1A2*

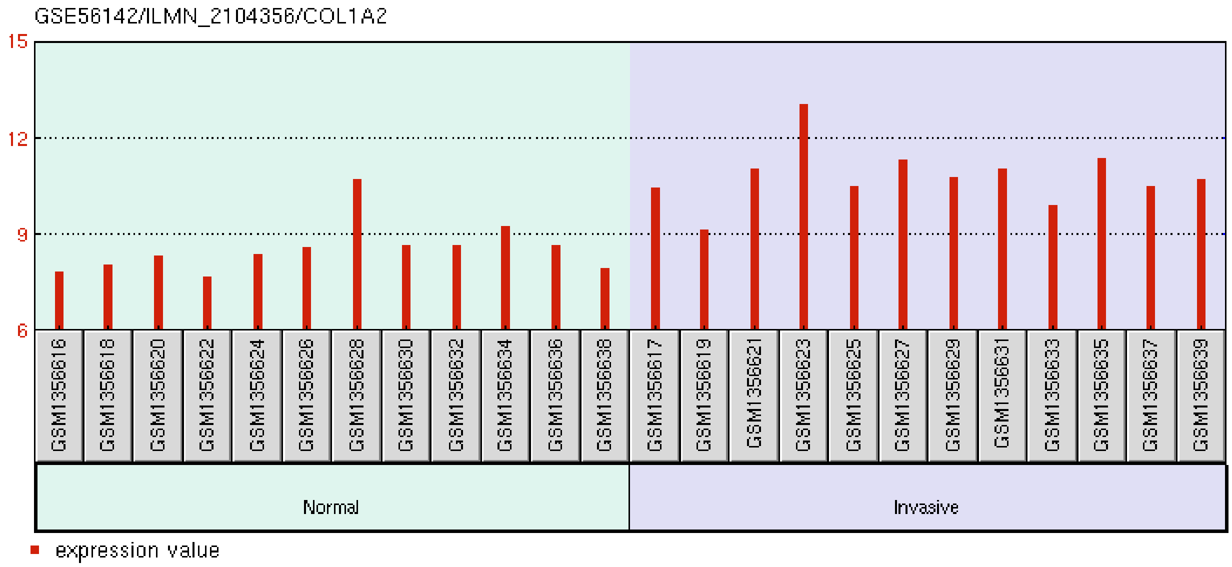


## *COL3A1*

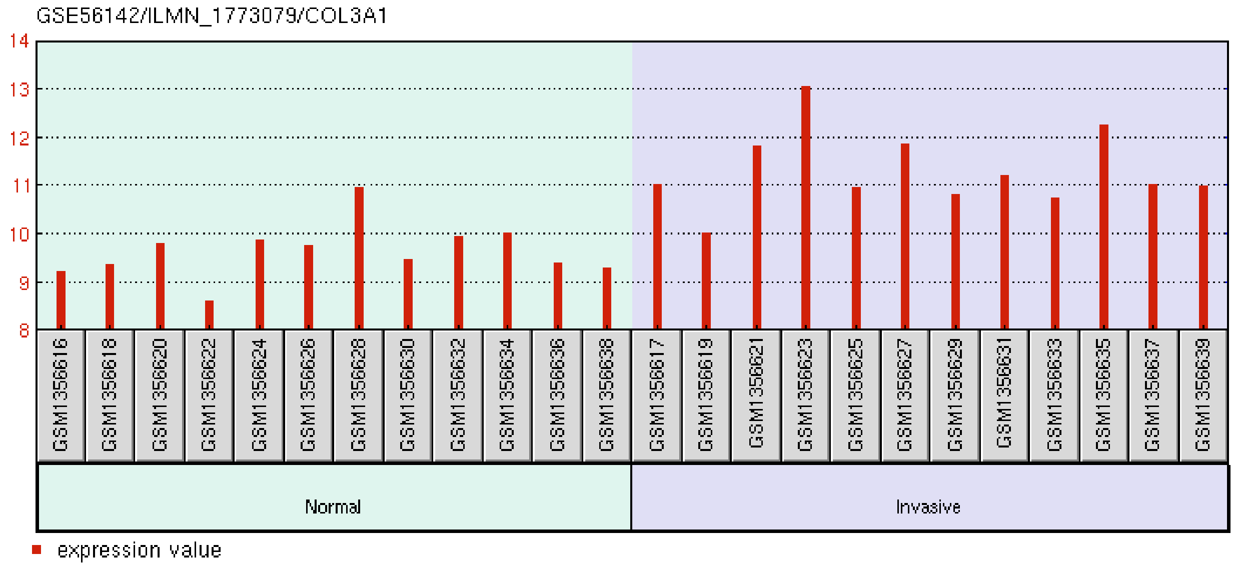

## *COL4A1*

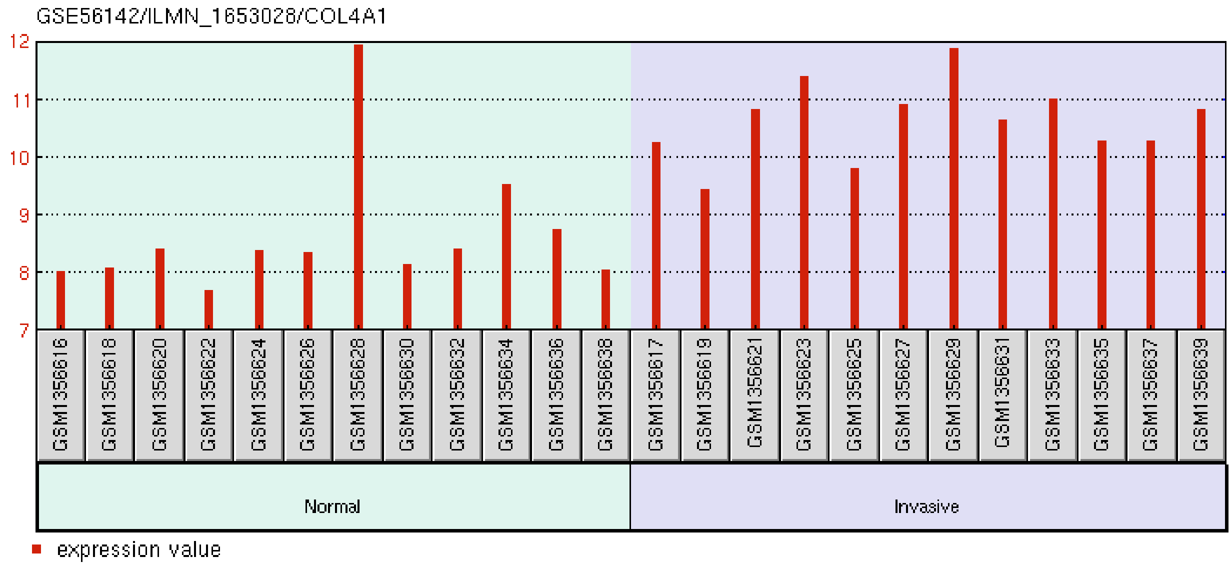


## *COL6A3*

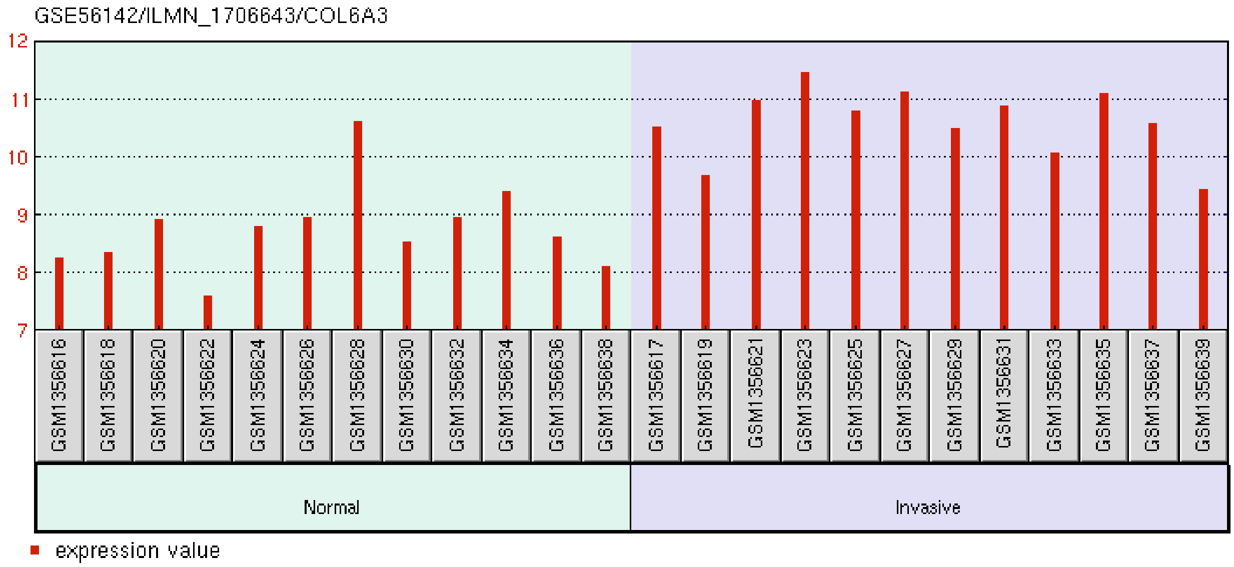

## *CRCT1*

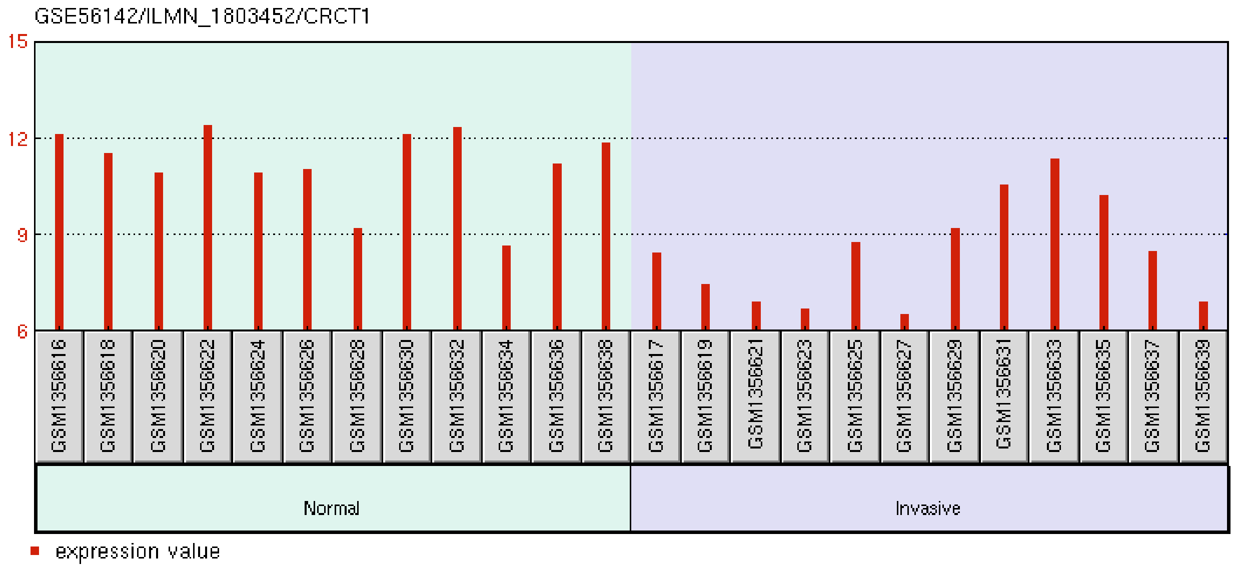
GSE56142/ILMN_1803452/CRCT1
15
12
9
6
GSM1356616
GSM1356618
GSM1356620
GSM1356622
GSM1356624
GSM1356626
GSM1356628
GSM1356630
GSM1356632
GSM1356634
GSM1356636
GSM1356638
GSM1356617
GSM1356619
GSM1356621
GSM1356623
GSM1356625
GSM1356627
GSM1356629
GSM1356631
GSM1356633
GSM1356635
GSM1356637
GSM1356639
Normal
Invasive
expression value

## *FAM3D*

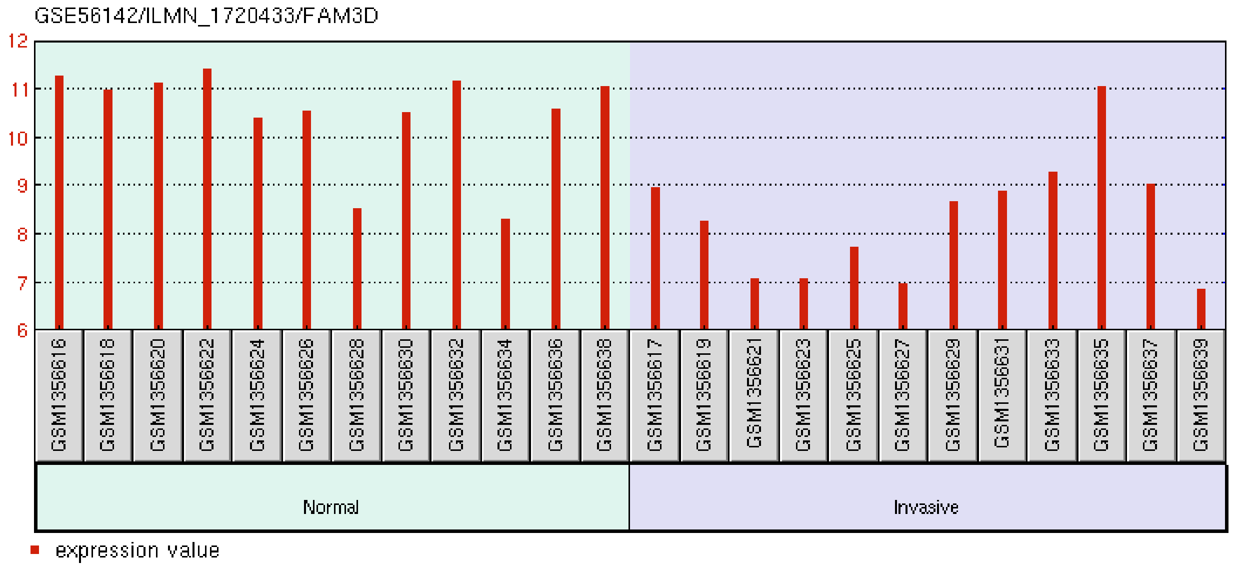
GSE56142/ILMN_1720433/FAM3D
12
11
10
9
8
7
6
GSM1356616
GSM1356618
GSM1356620
GSM1356622
GSM1356624
GSM1356626
GSM1356628
GSM1356630
GSM1356632
GSM1356634
GSM1356636
GSM1356638
GSM1356617
GSM1356619
GSM1356621
GSM1356623
GSM1356625
GSM1356627
GSM1356629
GSM1356631
GSM1356633
GSM1356635
GSM1356637
GSM1356639
Normal
Invasive
expression value

## *IFI6*

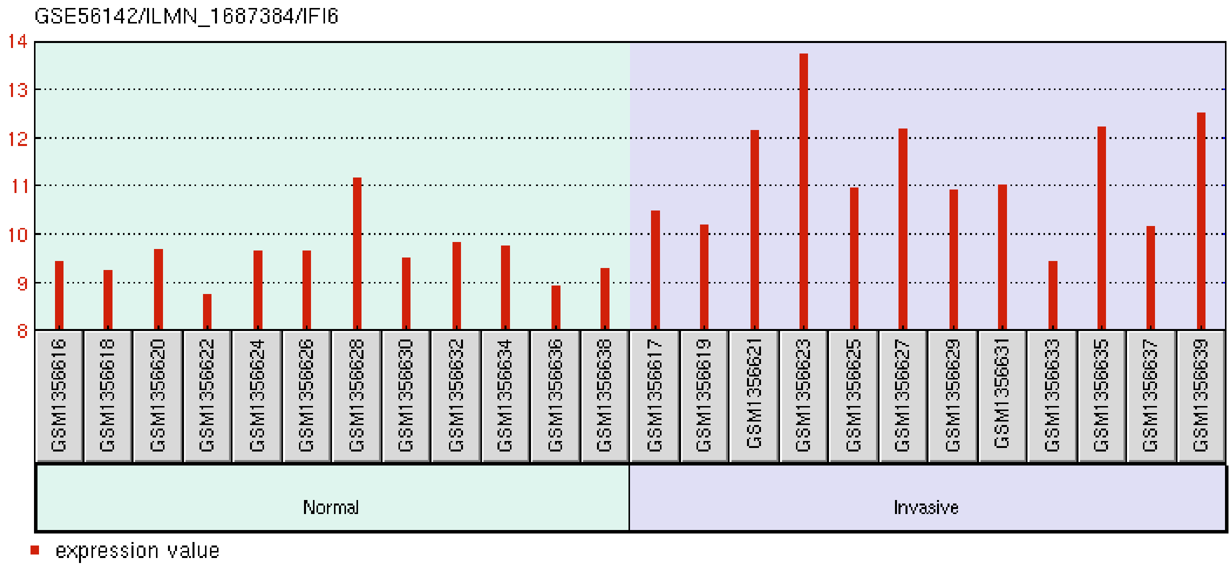
GSE56142/ILMN_1687384/IFI6
14
13
12
11
10
9
8
GSM1356616
GSM1356618
GSM1356620
GSM1356622
GSM1356624
GSM1356626
GSM1356628
GSM1356630
GSM1356632
GSM1356634
GSM1356636
GSM1356638
GSM1356617
GSM1356619
GSM1356621
GSM1356623
GSM1356625
GSM1356627
GSM1356629
GSM1356631
GSM1356633
GSM1356635
GSM1356637
GSM1356639
Normal
Invasive
expression value

## *KRT78*

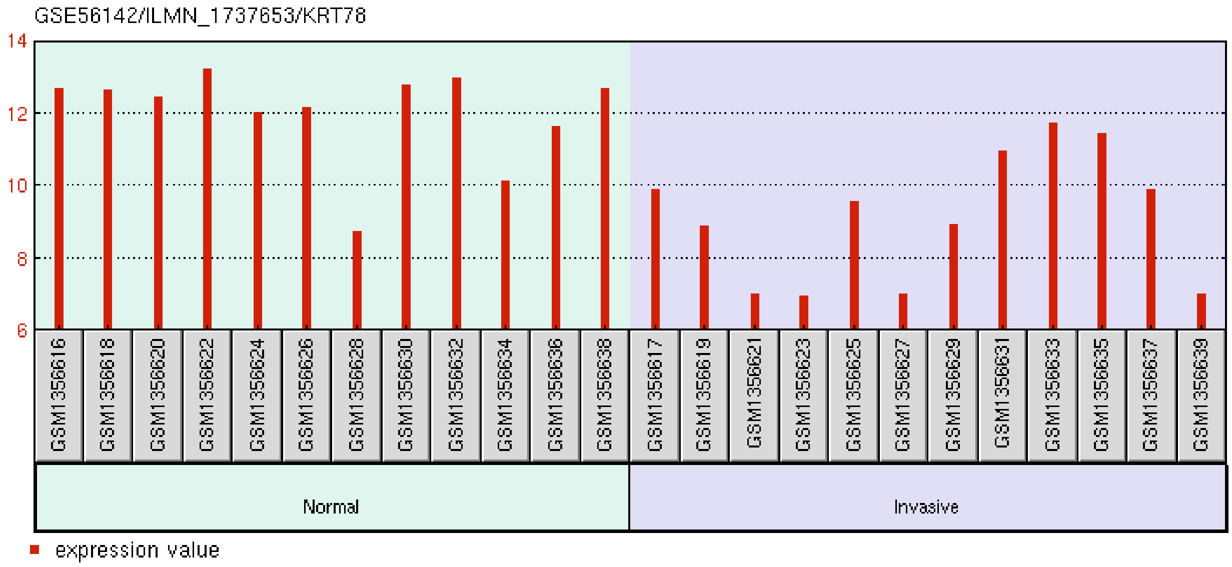
GSE56142/ILMN_1737653/KRT78
14
12
10
8
6
GSM1356616
GSM1356618
GSM1356620
GSM1356622
GSM1356624
GSM1356626
GSM1356628
GSM1356630
GSM1356632
GSM1356634
GSM1356636
GSM1356638
GSM1356617
GSM1356619
GSM1356621
GSM1356623
GSM1356625
GSM1356627
GSM1356629
GSM1356631
GSM1356633
GSM1356635
GSM1356637
GSM1356639
Normal
Invasive
expression value

## *MUC21*

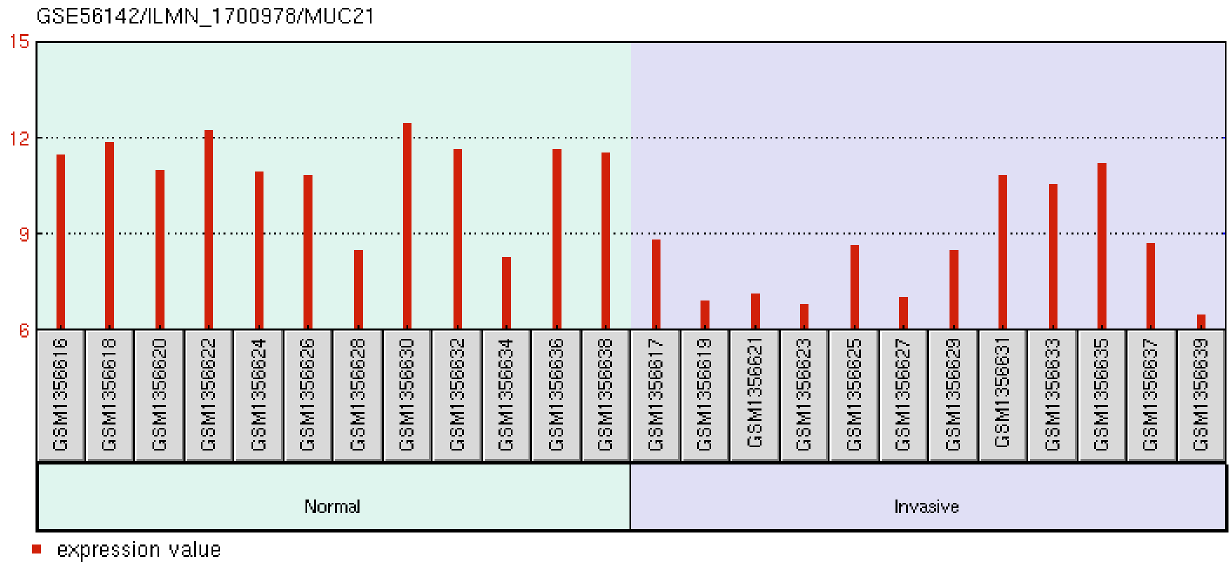
GSE56142/ILMN_1700978/MUC21
15
12
9
6
GSM1356616
GSM1356618
GSM1356620
GSM1356622
GSM1356624
GSM1356626
GSM1356628
GSM1356630
GSM1356632
GSM1356634
GSM1356636
GSM1356638
GSM1356617
GSM1356619
GSM1356621
GSM1356623
GSM1356625
GSM1356627
GSM1356629
GSM1356631
GSM1356633
GSM1356635
GSM1356637
GSM1356639
Normal
Invasive
expression value

## *NDRG2*

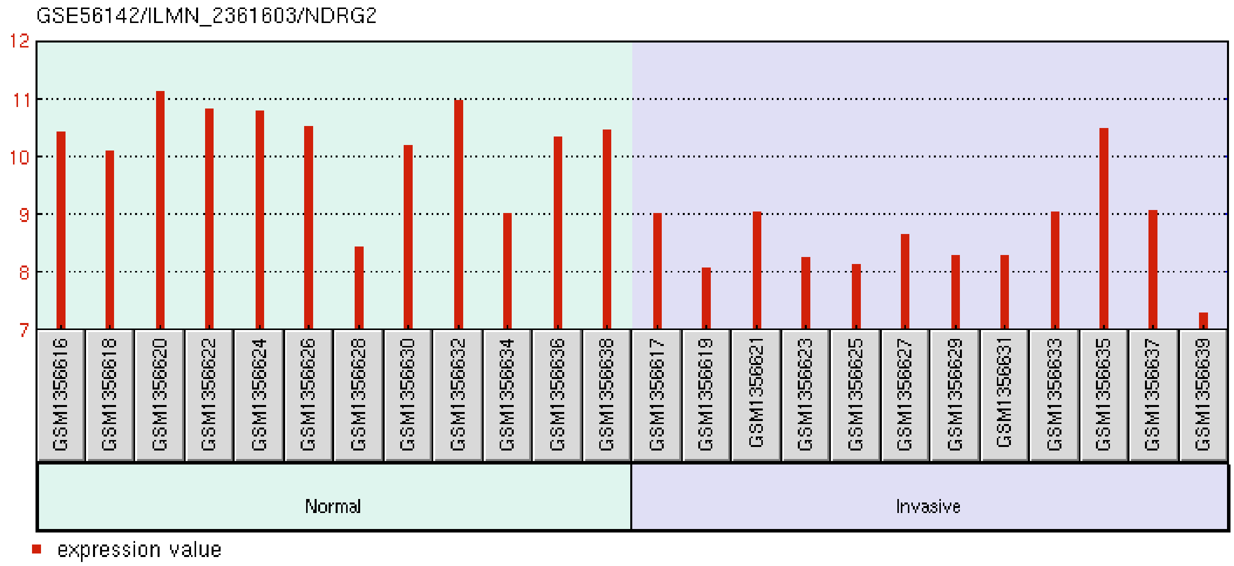
GSE56142/ILMN_2361603/NDRG2
12
11
10
9
8
7
GSM1356616
GSM1356618
GSM1356620
GSM1356622
GSM1356624
GSM1356626
GSM1356628
GSM1356630
GSM1356632
GSM1356634
GSM1356636
GSM1356638
GSM1356617
GSM1356619
GSM1356621
GSM1356623
GSM1356625
GSM1356627
GSM1356629
GSM1356631
GSM1356633
GSM1356635
GSM1356637
GSM1356639
Normal
Invasive
expression value

## *OSBPL10*

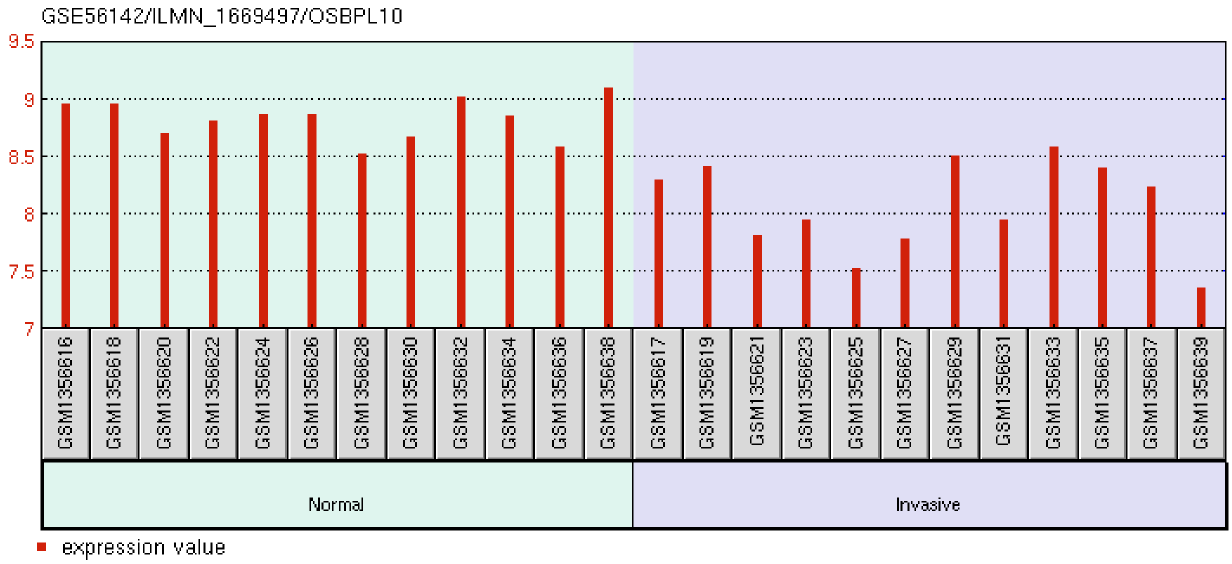


## *PDGFRB*

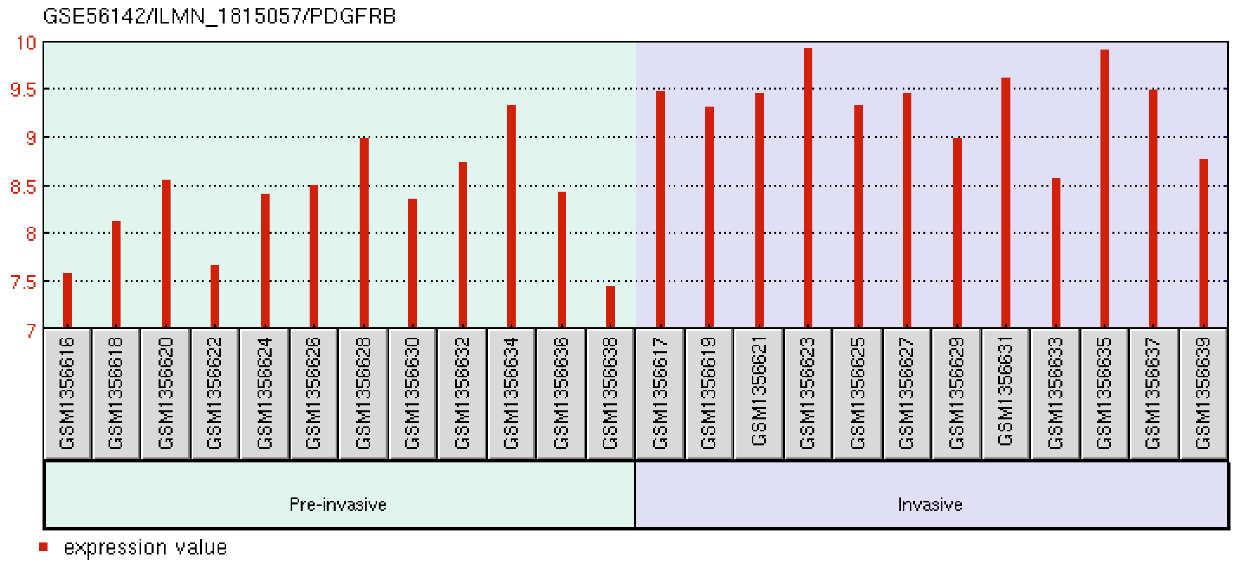

## *RCN1*

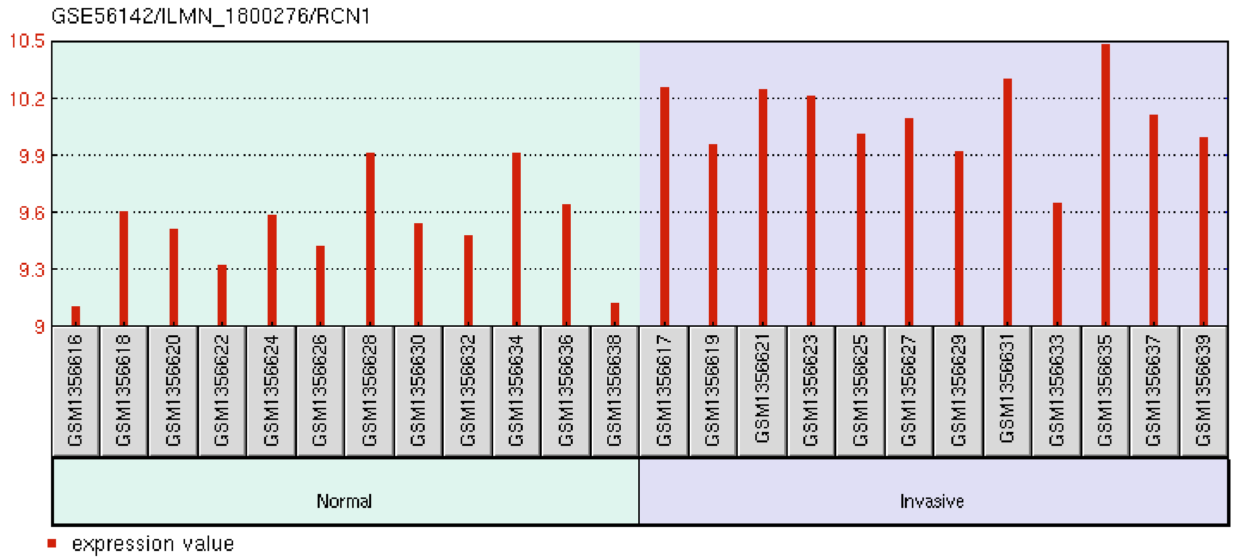
GSE56142/ILMN_1800276/RCN1
10.5
10.2
9.9
9.6
9.3
9
GSM1356616
GSM1356618
GSM1356620
GSM1356622
GSM1356624
GSM1356626
GSM1356628
GSM1356630
GSM1356632
GSM1356634
GSM1356636
GSM1356638
GSM1356617
GSM1356619
GSM1356621
GSM1356623
GSM1356625
GSM1356627
GSM1356629
GSM1356631
GSM1356633
GSM1356635
GSM1356637
GSM1356639
Normal
Invasive
expression value

## *RNASE7*

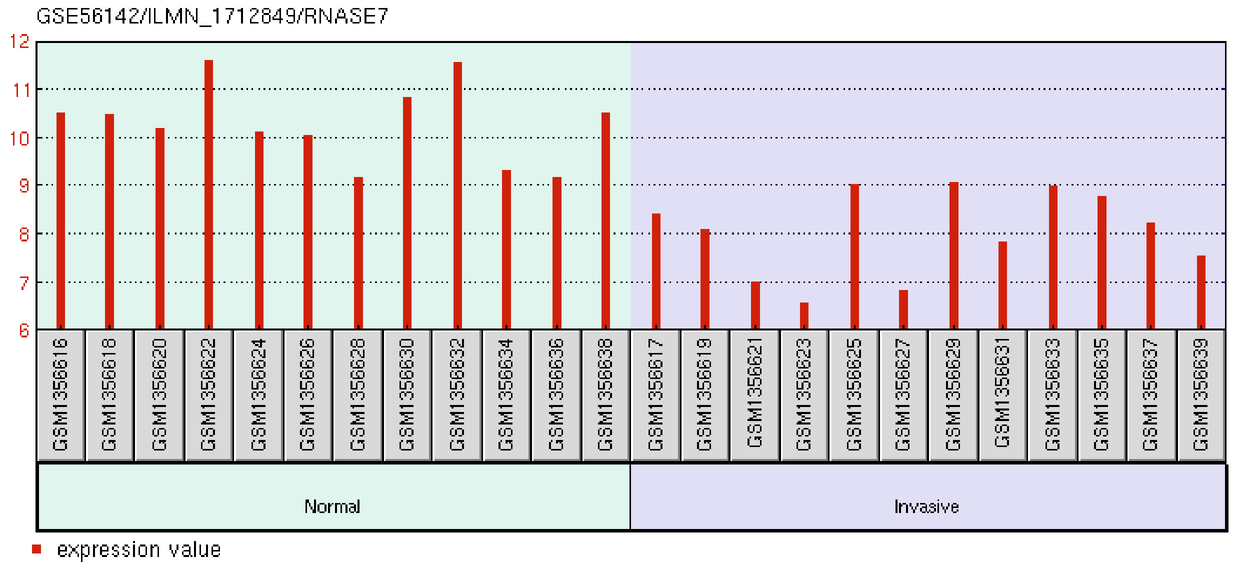
GSE56142/ILMN_1712849/RNASE7
12
11
10
9
8
7
6
GSM1356616
GSM1356618
GSM1356620
GSM1356622
GSM1356624
GSM1356626
GSM1356628
GSM1356630
GSM1356632
GSM1356634
GSM1356636
GSM1356638
GSM1356617
GSM1356619
GSM1356621
GSM1356623
GSM1356625
GSM1356627
GSM1356629
GSM1356631
GSM1356633
GSM1356635
GSM1356637
GSM1356639
Normal
Invasive
expression value

## *SLURP1*

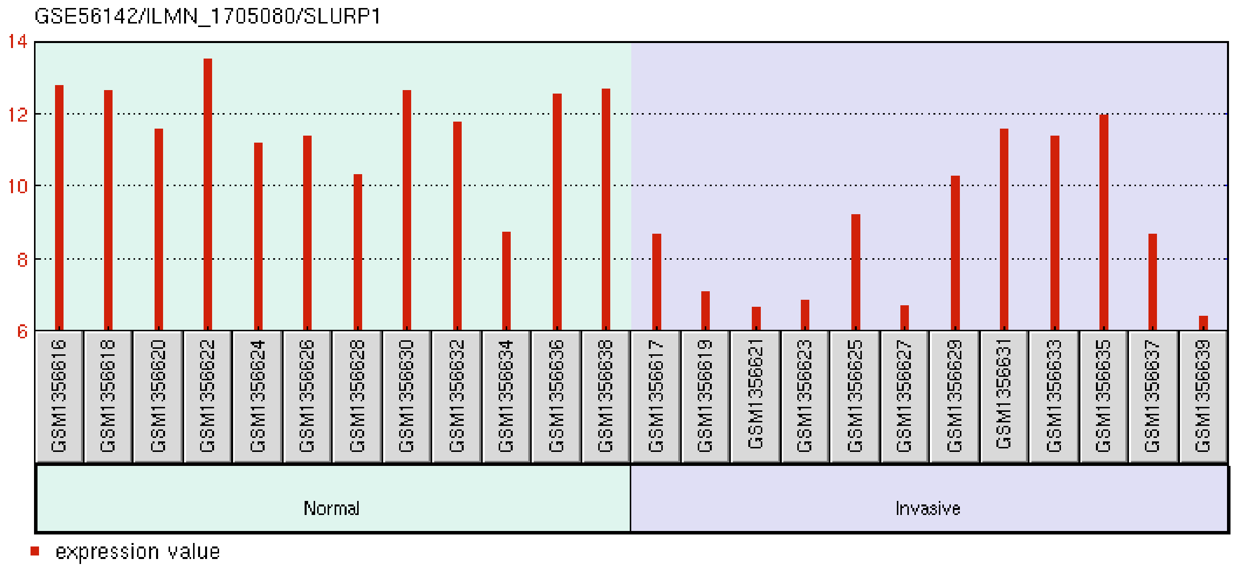


## *TEAD2*

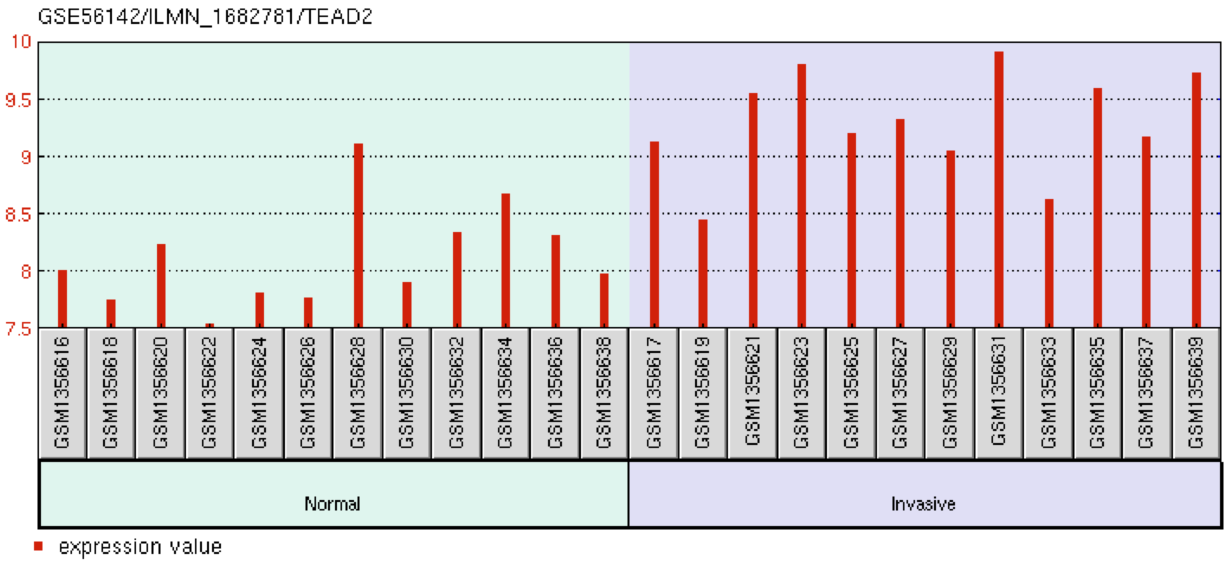

## *TYRO3*

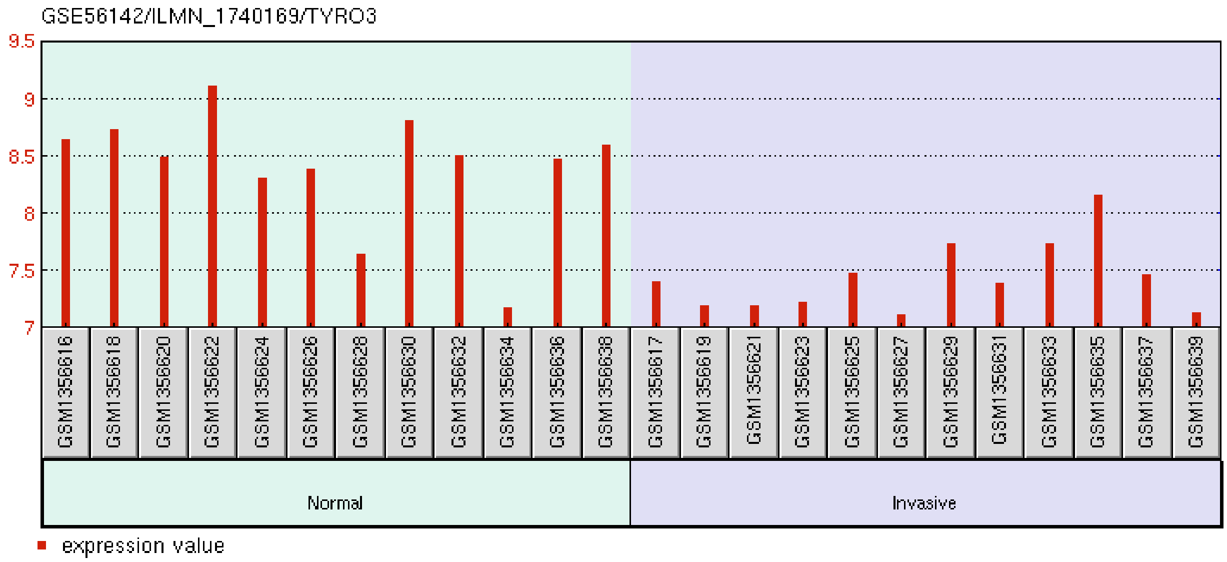

GSE56142/ILMN_1740169/TYRO3
9.5
9
8.5
8
7.5
7
GSM1356616
GSM1356618
GSM1356620
GSM1356622
GSM1356624
GSM1356626
GSM1356628
GSM1356630
GSM1356632
GSM1356634
GSM1356636
GSM1356638
GSM1356617
GSM1356619
GSM1356621
GSM1356623
GSM1356625
GSM1356627
GSM1356629
GSM1356631
GSM1356633
GSM1356635
GSM1356637
GSM1356639
Normal
Invasive
expression value
